# Intelligence

**Published:** 1808-07

**Authors:** 


### INTELLIGENCE.

Mr. Davy, in the concluding Lectures of his Course at the Royal Institution, gave a distinct and very luminous exhibition of his grand discovery, the decomposition of the alkalies. His first experiments were with potash and soda, which in their dry state are non-conductors, but when moistened, they have the property of conducting electricity. In both instances he clearly produced metalline substances, by bringing them within the action of the Voltaic battery. Oxygen was given out at the positive side of the battery, and at the negative little globules of the metallic base were instantly formed. The metalline bases of the alkalies he named Potassium and Sodaium, chusing the termination *um*, in compliance with the present nomenclature of metals, in order that they might agree with Platin*um*, Plumb*um*, &c. &c. These new metals, to appearance, are precisely like mercury, but very different from that metal in their various properties, which he enumerated and demonstrated to the satisfaction of every one present. He shewed the great inflammability of the new metals, by touching them with the smallest quantity of water, when they instantly took fire. They are both malleable at the common temperature, and may be spread into very thin leaves on a plate of glass, by mere pressure. So great, however, is their attraction for the oxygen of the atmosphere, that they almost instantly became tarnished. Before the discovery of these new metals, only two bodies of this class, viz. iron and platinum, were capable of being welded, and that at a very great heat, whereas separate parts of the Potassium and Sodaium can be united readily at the common temperature of the atmosphere. The specific gravity of the Potassium, is to that of water as about six to ten; in the case of sodaium, it is as ninety-three to one hundred. Mr. Davy shewed, that the metals which he had spread on glass, were easily fusible again by the heat of a spirit lamp, by the application of which they almost instantly run into globules. The attraction of these new metals for oxygen, was shewn by their burning at a red heat, when the alkalies were again revived. In analyzing them with great accuracy, he found the potash contained fourteen parts of oxygen and eighty-four of metalline base, but the soda contained twenty parts of oxygen to eighty of base. These metals introduced into a jar of oxymuriatic gas spontaneously took fire, and the white fume collected on the glass was in the one case muriate of potash, in the other muriate of soda, or common culinary salt. Particles of the new metals put into water, decomposed the water, and gave out a brilliant light. The water used was pure distilled water, which instantly after the decomposition of the metals, shewed by means of turmeric paper, that it was become highly alkaline. The same sort of action was exhibited by the nitric acid; and the potassium acted also on the sulphuric and other strong acids. Mr. Davy placed small particles of the metals on ice, and they immediately decomposed the water and gave out a bright light: alkali was likewise formed on the ice.

These metals unite with mercury and form solids; thus, one part of sodaium and three of mercury being mixed, the two fluids united in a state of solidity. Several other experiments were made, and the Professor felt no hesitation in giving his opinion as to the great importance of these discoveries in their application to the arts; and even to an art, which he doubted if he should venture to mention, the art of war, as the destruction occasioned by these metals, if they could be brought into action, would be considerably greater than by those now in use. In reference to the detail of his own discoveries, he said the present state of knowledge was more likely to produce in him humility than exultation. The experimentalist was not the hero of his own tale, but he could not avoid being the subject of it; and he hoped the experiments and facts exhibited, would be considered independently of any opinion which he had divulged in connection with them. In the present imperfect state of enquiry, it would be presumption to expect that any thing would be permanently established. Alluding to the great powers of the Voltaic battery,* he said, some might imagine that the desideratum of the Alchemists was accomplished, and that the delusive hopes of those visionaries were, at length, realized: but it ought to be remembered there was an immense interval betwen the processes of combination and decomposition; that the proper province of chemisty extended but to inorganic matter; that in the powers of combination other agents were employed, with whose nature we were wholly unacquainted, and to whose operation we might for ever remain strangers.

Mr. Davy, having shewn his experiments on the fixed alkalies, proceeded to Ammonia, which, from the manipulations of Priestley and Steele, was regarded as a compound of Hydrogen and Nitrogen: he had, no doubt, in his mind, that it contained also a portion of oxygen. This he attempted to demonstrate by experiments, particularly by passing some ammonia, in a state of gas, through a porcelain tube, heated to a white heat; the hydrogen and nitrogen were collected, and in the glass vessel through which they passed, moisture was very apparent, which he felt satisfied was water, formed by the oxygen contained in the ammonia. Hence he inferred, that though oxygen had hitherto been considered as the acidifiable principle only, it would be found to be the alkalizing principle likewise.

From the alkalies he proceeded to the earths, which he enumerated

* Mr. Davy's battery contains thirty-eight thousand square inches of metal plates.

rated and described, and which he considered as the link between the alkalies and metallic oxides; and he had no doubt that they would hereafter yield to some higher powers of the Voltaic battery, and exhibit the parts of which they were composed. On Barytes he then made an experiment, shewing very decisively that it contained an inflammable principle. He was next led to consider the phænomena of meteoric stones; and the light occasioned by them; Mr. Davy adds, were now perfectly explicable by the facts just discovered; but as to the place whence these bodies came, he gave no opinion, only that from the curves which they described, it was certain that they came from some other world, and were travellers only in our atmosphere; for if they had been formed there, their descent must be perpendicular to the surface of the earth, which, it was known, was not the case. The Professor then referred to the several substances that had hitherto been deemed simple; supposing that all might, hereafter, be decomposed; in sulphur and charcoal, it was now known there was hydrogen; he seemed to suspect that the two great principles operating in nature, were the principle of inflammability and a metallic principle.—It is our intention in a subsequent Number, to give a more detailed account of the discoveries of this learned chemist, which cannot fail to be highly interesting to every lover of science, and more particularly to those who having attended the lectures at the Royal Institution, will be glad to have the facts and experiments there exhibited, brought again to their recollection.

---

M. Le Lievre, a member of the national Institute of France, has discovered in the Island of Elba a new mineral, to which he has given the name of Jenite, in honour of the battle of Jena. It is opaque, of a black colour, sometimes inclining to brown, and is nearly four times as heavy as distilled water. From the analysis of Messrs. Vauquelin and Descotils, it appears that this mineral contains rather more than half its weight of iron, mixed with a little manganese, and that the rest of the stone is lime and silex, the proportion of the silex being considerably more than double that of the lime.

Dr. Joseph Reade has published an Account of an Experiment, the result of which is in direct contradiction to the received opinion, that the agitation or friction of fluids cannot excite sensible heat. It is as follows:—The temperature of the apartment being 40°, half a pint of water at a similar heat, was poured into a tin bottle-shaped vessel, into the aperture of which was inserted a thermometer surrounded with chamois leather, and made to fit accurately, with its bulb nearly in the axis. After briskly agitating the vessel for a few minutes, to his great surprise he found that the temperature of the water rose eight degrees; and, even after the apparatus was uncovered and laid at rest on the table, the water continued to rise for several minutes; proving the origin of the heat to be inherent in the fluid, and independent of any external causes. Anxious to obviate every source of fallacy or objection, ion, Dr. Reade prevented the communication of caloric by his hands, or of radiation from his body, by coating the tin vessel with many layers of woollen cloth carefully wrapped round it, over which there was a tin case, the entire nearly two inches in thickness, and covered externally with three wet towels. In the course of the experiment he dipped his hands frequently in snow water, and also sprinkled the towels. The Rev. Mr. Hincks, lecturer on chemistry in the Cork Institution, to whom the experiment was communicated, on repeating it in a glass bottle, found the heat of the vessel, by means of a thermometer placed between it and the covering, to be inferior to that of the enclosed fluid, and on a par with the atmosphere, which proves in a most satisfactory manner that there could be no communication of caloric from the hand.

According to some cruel, though interesting, experiments, reported to the French Institute by M. Dupuytren, principal Director of Anatomy at the Medical School, and M. Duprey, Professor at the Veterinary School at Alfort, on the subject of respiration; it appears, that the section of the two nerves of the eighth pair (those of the stomach and breast) in animals, is certain and instant death; and that respiration, the most important function of life, is exercised directly under the power of nervous influence, and is inseparable from it.

In the course of this month will be published, a Supplementary Volume of Birds to Barr's Edition of Buffon. From the splendid edition of Buffon's Works, with additions, lately published by Sonnini, has been selected every article of importance or of curiosity; and several new plates of rare birds will accompany this volume, the contents of which will bring down the era of discovery in this interesting branch of Natural History to the present day.

Dr. George Alley, of Cork, has nearly ready for the press, Observations on the Hydrargyria, or that peculiar species of eruptive disease which arises from the exhibition of mercury; to be illustrated with coloured engravings. This publication will contain all the information on this singular and interesting disease, which the observations of those gentlemen whose attention has been particularly directed to the subject, have afforded; besides what the author was enabled to collect during an attendance of more than six years on the Westmoreland Lock Hospital, Dublin.

Dr. Reid, the author of the Reports of Diseases inserted regularly in the Monthly Magazine, intends to collect those which have appeared hitherto, into a small volume, to be published early in the winter, printed uniformly with his Treatise on Consumption.

